# Thyrotropin Receptor Epitope and Human Leukocyte Antigen in Graves’ Disease

**DOI:** 10.3389/fendo.2016.00120

**Published:** 2016-08-23

**Authors:** Hidefumi Inaba, Leslie J. De Groot, Takashi Akamizu

**Affiliations:** ^1^The First Department of Medicine, Wakayama Medical University, Wakayama, Japan; ^2^Department of Cellular and Molecular Biology, University of Rhode Island, Providence, RI, USA

**Keywords:** TSH receptor, HLA, Graves’ disease, epitope, anti-TSHR-antibody

## Abstract

Graves’ disease (GD) is an organ-specific autoimmune disease, and thyrotropin (TSH) receptor (TSHR) is a major autoantigen in this condition. Since the extracellular domain of human TSHR (TSHR-ECD) is shed into the circulation, TSHR-ECD is a preferentially immunogenic portion of TSHR. Both genetic factors and environmental factors contribute to development of GD. Inheritance of human leukocyte antigen (HLA) genes, especially HLA-DR3, is associated with GD. TSHR-ECD protein is endocytosed into antigen-presenting cells (APCs), and processed to TSHR-ECD peptides. These peptide epitopes bind to HLA-class II molecules, and subsequently the complex of HLA-class II and TSHR-ECD epitope is presented to CD4+ T cells. The activated CD4+ T cells secrete cytokines/chemokines that stimulate B-cells to produce TSAb, and in turn hyperthyroidism occurs. Numerous studies have been done to identify T- and B-cell epitopes in TSHR-ECD, including (1) *in silico*, (2) *in vitro*, (3) *in vivo*, and (4) clinical experiments. Murine models of GD and HLA-transgenic mice have played a pivotal role in elucidating the immunological mechanisms. To date, linear or conformational epitopes of TSHR-ECD, as well as the molecular structure of the epitope-binding groove in HLA-DR, were reported to be related to the pathogenesis in GD. Dysfunction of central tolerance in the thymus, or in peripheral tolerance, such as regulatory T cells, could allow development of GD. Novel treatments using TSHR antagonists or mutated TSHR peptides have been reported to be effective. We review and update the role of immunogenic TSHR epitopes and HLA in GD, and offer perspectives on TSHR epitope specific treatments.

## Introduction

**Figure 1:**
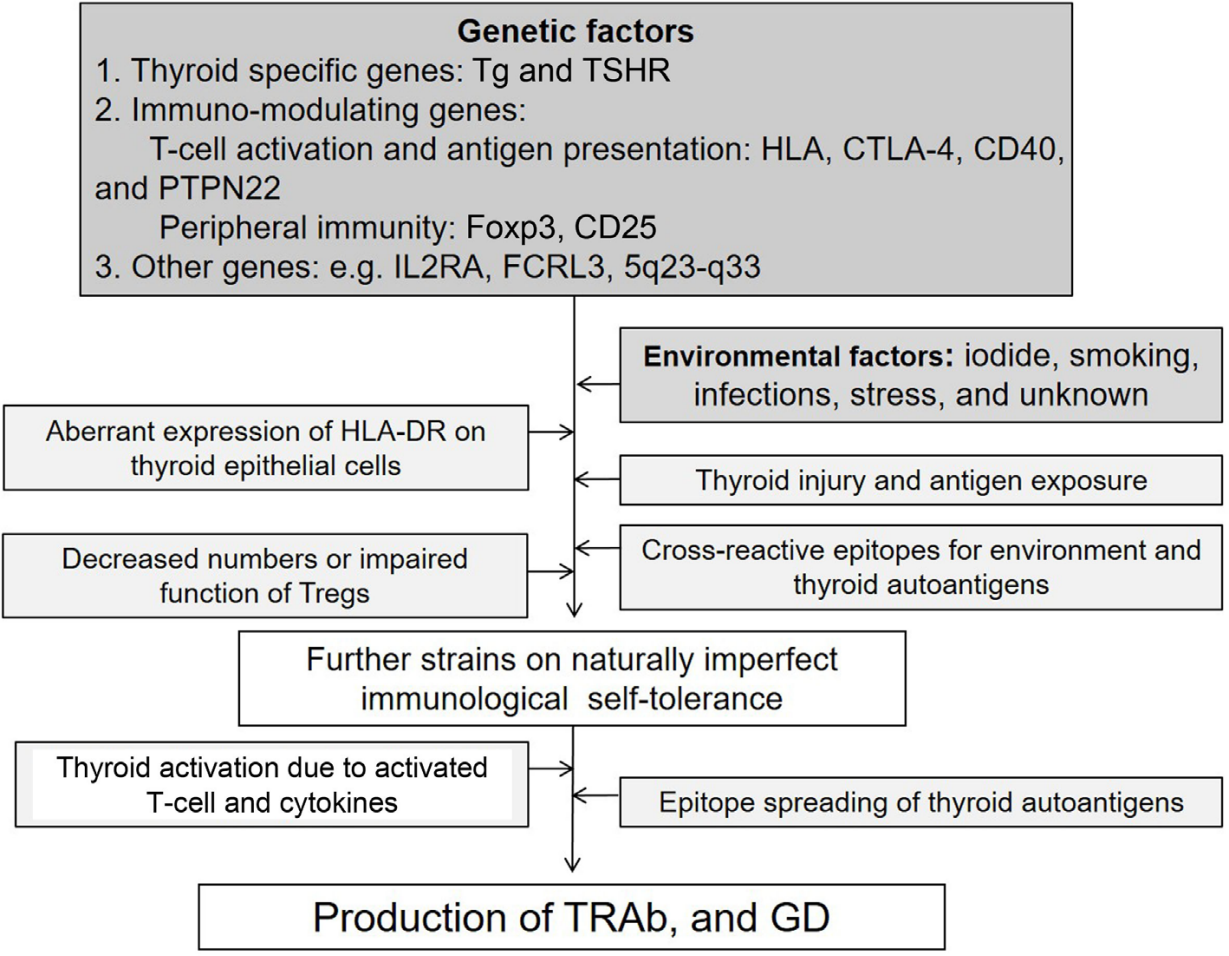
**Factors possibly contributing to the etiology of Graves’ disease (GD)**.

**Figure 2:**
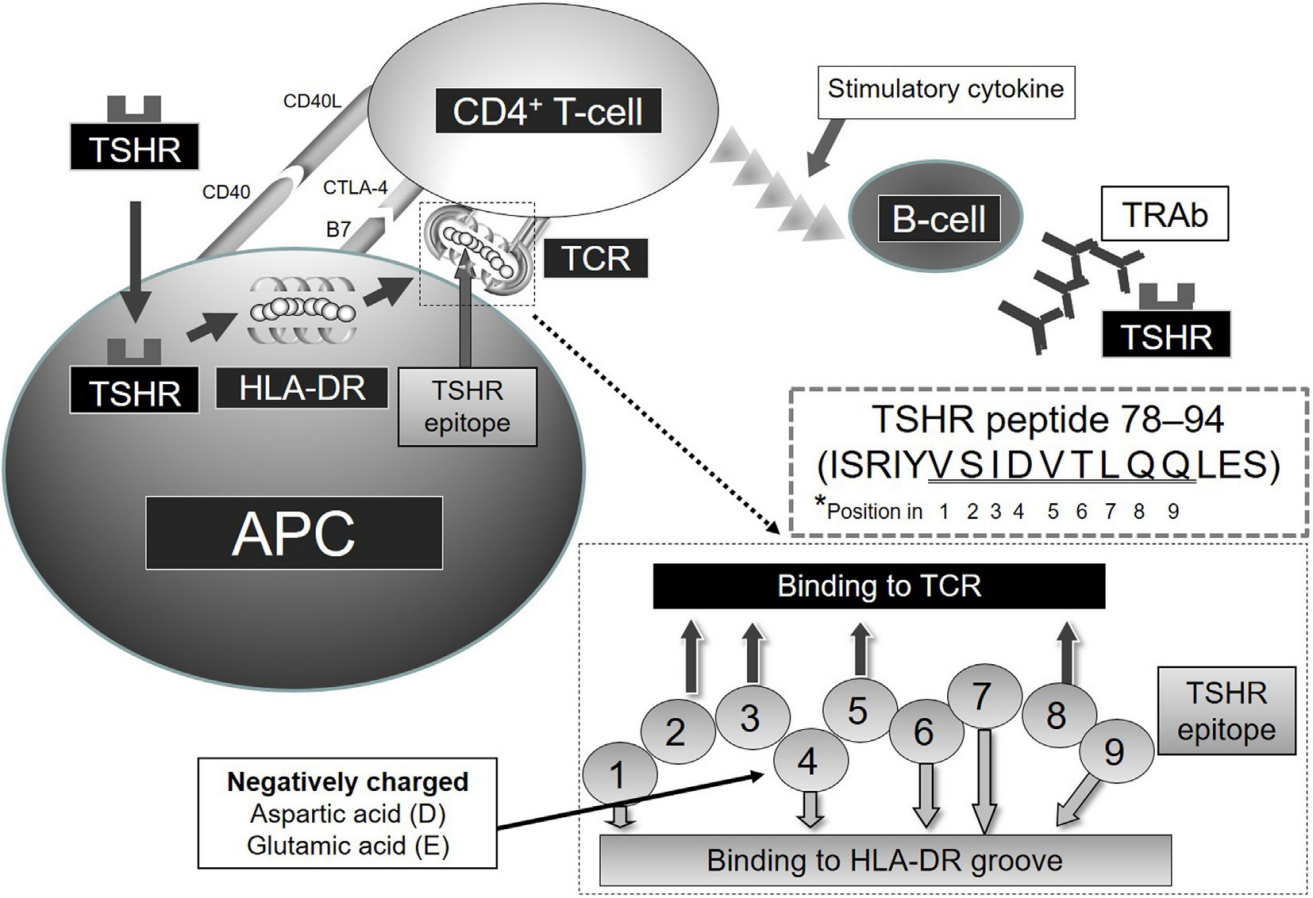
Relation of TSHR and HLA-DR in GD *Note that amino acids of TSHR epitope 78–94 (underlined residues VSIDVTLQQ) are predicted to contact the HLA-DR-binding groove or TCR at positions 1–9, respectively.

Autoimmune thyroid diseases (AITDs) are organ-specific autoimmune diseases with multiple etiologies (1) (Figure 1). Graves’ disease (GD) and Hashimoto’s thyroiditis (HT) are two major components of AITDs. When individuals having susceptible genetic background are exposed to environmental factors (e.g., iodine, smoking, infections, and stress, and others so far undisclosed), thyroid autoantigens break “self-tolerance” and AITDs develop (2). Thyroid autoantigens, such as thyroglobulin (Tg), thyrotropin (TSH) receptor (TSHR), thyroid peroxidase (TPO), and NIS have increased immunogenicity when they are iodinated, and glycosylated. Tg and TSHR have genetic polymorphisms that may predispose to GD (1). Specific polymorphisms of other genes [e.g., human leukocyte antigen (HLA), cytotoxic T-lymphocytes antigen (CTLA-4), CD40] are clearly associated with GD (3–6). GD is characterized by hyperthyroidism caused by stimulatory anti-TSHR antibodies (TRAb, TSAb, TSI) (7). TSHR peptide epitopes bound to HLA-class II are presented by antigen-presenting cells (APCs) to CD4+ T cells (Figure 2). Interaction by the complex of TSHR epitope, HLA-class II molecule, and T-cell receptor (TCR) is modified through binding of CD40 ligand to CD40 and of CTLA-4 to B7 (3–6). TSHR epitopes bound to HLA-class II presented on the surface of APC are the most crucial factor to determine immunogenicity. Various approaches to identify the TSHR epitopes have involved *in silico, in vitro, in vivo*, and clinical studies, and some TSHR epitope clusters were reported (7, 8). Thyroid function is regulated by not only TRAb but also cell-mediated immunity (9). Two major regulations (central and peripheral) maintain self-tolerance (2). We review the immunogenic mechanisms of GD in association with TSHR and HLA, and discuss future therapeutic approaches.

## TSHR and GD

TSH receptor is one of a family of glycoprotein-coupled hormone receptors, and was cloned in 1990 (10). TSHR is indispensable for TSH signal transduction, production of thyroid hormone and Tg, and proliferation of thyroid follicular cells. TSHR consists of an extracellular domain (ECD: amino acids 1–418), a seven transmembrane domain (7TMD: 418–683) and an intracellular domain (11). ECD is also divided into Leucine-rich repeat domain (LRR: 1–276) and a hinge region (277–418). The region around 7TMD is referred to as serpentine domain (11). Upon TSHR activation, TSH or TRAb binds to TSHR, and signal is transduced through 7TMD into Gαs. Recently, Brüser et al. found that a peptide named P10 (TSHR-405-FNPCEDIMGY-414) located in C-terminus of TSHR-ECD, is conserved in different GPHRs-ECD and different species. They found that P10 can activate TSHR *in vitro*, and suggested that P10 isomerizes and induces structural changes in the 7TMD, triggering Gαs activation (12) on TSHR-ECD ligand binding. Schaarschmidt et al. proposed that the re-arrangement of the ECD (extracellular loop 1) was critical for TSHR activation (13). TSHR is alternatively divided into an A-subunit (amino acids 1–302), C-domain (303–367), and B-subunit (368–764) (14). Deletion of 50 amino acids in C-domain (residues 317–366) had no effect on TSH binding or on TSH and TSAb-stimulating activities (15). After C-domain is physiologically cleaved (15), A-subunit (residues 22–289) is shed into the circulation (14). Thus, TSHR A-subunit is thought to be preferentially immunogenic in GD (16), and also in animal GD models (17). Importantly, two portions in TSHR A-subunit (246–260 and 277–296) and another region in TSHR B-subunit (381–385) fold together to form a complex TSH-binding pocket (18).

## Genetic Factors in GD

In research on twins, genetic factors were found to contribute 79% to the likelihood of having GD (19). In a Japanese nationwide study in 1999, 2.1–3.1% of hyperthyroidism seemed to be familial, and the relative risk of familial GD was increased from 19- to 42-fold (20). Tomer and Davies reported that 33% of siblings of AITD patients developed AITD themselves, and 56% of siblings of AITD patients produced thyroid autoantibodies, also supporting a strong genetic influence on development of AITD (21). Genetic factors reported to predispose to GD include specific polymorphisms of HLA (3), CTLA-4 (4, 5), CD40 (6), protein tyrosine phosphatase-22 (PTPN22) (22), FOXP3, and CD25 (3). In addition, polymorphisms of TSHR (3, 4), Tg (3), interleukin-2 receptor alpha (IL2RA) (23), and Fc receptor-like3 (FCRL3) (24) were reported. Among these, HLA is a major genetic factor in AITD (3). The HLA locus is located on chromosome 6p21, and encodes (1) class I genes, such as HLA antigens A, B, and C, and (2) class II genes, such as HLA-DP, DQ, and DR genes (25). Inheritance of HLA-DRB1*03:01 (DR3) has been demonstrated to induce the highest susceptibility to GD in several ethnic groups (26, 27), and also in HT (3, 28). HLA-B8 was reported to be associated with GD in many studies (21). HLA-DQA1*05:01 was also reported to predispose to GD in Caucasians (26, 29). By contrast, HLA-DRB1*07:01 (DR7) was reported to be a protective allele for GD (30). The DR3 and DR7 alleles differ at the 74th amino acid in HLA-DRβ1, a critical residue in the binding pocket of the HLA-DR protein. The amino acid is arginine or glutamine, respectively. When DRB1*03:01 and DR7 alleles coexist, DR7 appears to suppress the susceptibility to GD conferred by DR3 (3). The HLA-genes have also been shown to be associated with GD in non-Caucasian populations, although the predisposing alleles are different from those observed in Caucasians. Chen et al. found that HLA-B*46:01, HLA-DPB1*05:01, HLA-DQB1*03:02, HLA-DRB1*15:01, and HLA-DRB1*16:02 were associated with GD in Taiwan (31). Recent studies in Japan have shown associations of GD with HLA-B*35:01, HLA-B*46:01, HLA-DRB1*14:03, and HLA-DPB1*05:01 (32). These authors reported that the protective allele, HLA-DRB1*13:02 overwhelms the GD-susceptibility of DP5 when they coexist. Many other gene associations have been reported. Vita et al. recently reported that certain HLA alleles are associated with stress-triggered GD and with clinical outcomes (33). The second most important gene polymorphism involves CTLA-4, which is expressed on activated T cells. It binds to B7 on the surface of APC to suppress T-cell-mediated immunity through co-suppressive signals (4).

One group has consistently reported association of TSHR gene polymorphisms with GD in Japanese (3, 4, 34). A Tg polymorphisms, in association with DR3, is also considered to relate to GD (3). Tomer et al. reported interaction between a Tg gene variant and DRB1-Arg 74 in predisposing to GD, increasing the odds ratio to more than 36 (28). Furthermore, they confirmed that TSHR, CTLA-4, and Tg genes are associated with GD in Italians (35). In an age-related aspect, Brown et al. identified novel susceptibility loci related to young age onset of GD (36). In a mouse model of GD, TRAb were genetically linked to both MHC-class I and Class II antigens (37).

Recently, Limbach et al. found hypermethylation of T-cell signaling genes and TSHR gene, suggesting dysregulation in T cell and TSHR signaling in GD patients (38). Stefan et al. also reported a genetic–epigenetic interaction involving a non-coding SNP in the TSHR gene that controls thymic TSHR gene expression and promotes escape of TSHR-reactive T cells from central tolerance, triggering GD (39).

## Relation of TSHR-ECD and HLA-DR

Shed TSHR-ECD protein is endocytosed into APCs and processed to TSHR-ECD peptides in lysosomes (Figure 2). These peptide epitopes bind to HLA-DR molecules, and subsequently the complex of HLA-DR and TSHR-ECD epitope is presented on APCs to CD4+ T cells. Aberrant expression of HLA-DR molecules was first considered as a trigger of GD (40), and later both TSHR and HLA-DR were found to be critical in the process of autoimmunity in GD (41). Recombinant human interferon (IFN)-α was reported to increase the expression of HLA-DR and TSHR on thyrocytes in GD subjects and not in controls (42). Shimojo et al. first reported that fibroblasts co-transfected with both human TSHR and MHC-class II could induce GD in mice (43). Lymphocytes infiltrating the thyroid in human TSHR A-subunit transgenic mice are involved in recognition of human TSHR A-subunit by T cells activated using adenovirus encoding the human TSHR (44). The complex of TSHR-ECD epitope presented on APC with MHC-class II and recognition by T cells appears to be necessary to initiate an immunogenic reaction.

We examined the binding affinity between TSHR-ECD epitopes and HLA-DR *in silico, in vitro*, and *in vivo* studies (7, 8, 45–47). Predicted binding affinities of TSHR-ECD peptides to epitope-binding groove in various HLA-DRs were examined using computer algorithms (7). These studies *in silico* and *in vitro* showed the priority of strong binders to HLA-DR in terms of immunogenicity. The peptide-binding groove in HLA-DR consists of nine amino acids. Amino acids in positions 1, 4, 6, 7, and 9 bind to HLA-DR and those in positions 2, 3, 5, and 8 face the TCR. We found that the amino acid position 4 of the amino acid sequence in the binding groove of HLA-DR is critical in determining binding affinity between the TSHR epitopes and HLA-DR (8). Positively charged Arginine in position 4 of the amino acid sequence in the binding motif of HLA-DRB1*03:01 appears also important (3, 8). TSHR-ECD epitopes with negatively charged D (aspartic acid) or E (glutamic acid) in position 4 of the binding motif bind more strongly to HLA-DR3 and are more stimulatory to GD patients’ peripheral blood mononuclear cells and to splenocytes from HLA-DR3 mice immunized to TSHR-ECD (9). As a result, TSHR-ECD peptide 132–150 (GIFNTGLKMFPDLTKVYST) was identified *in silico, in vitro*, and in clinical assays as an important epitope in GD, and peptide 78–94 (ISRIYVSIDVTLQQLES) was also identified as an important epitope when additional peptides were synthesized and used for assay as candidate epitopes (7, 45) (Figure 2). The possible importance of TSHR epitopes having moderate binding affinities to HLA-DR3; residues 145–163, 158–176, 207–222, 248–263, 272–291, and 343–362 was also identified (7). These epitopes appear important in immunogenicity to TSHR due to their favored binding to HLA-DR3, thus increasing presentation to T cells (8, 45).

T- and B-cell responses to genetic immunization differ in DR3 and DR2 transgenic mice. Mice transgenic for HLA-DR3 were more prone to develop AITD than were HLA-DR2 transgenic mice (45, 48). Pichurin et al. reported that in DR3 transgenic mice immunized to adenovirus coding TSHR 1–289, TSHR peptide (142–161) that is close to one of the epitopes mentioned above appeared to be a major T-cell epitope (49). Other groups also defined the T-cell epitopes in development of GD (50–53). Martin et al. found TSHR peptides 52–71, 142–161, 202–221, and 247–266 to be frequently recognized by CD4+ T cells from patients with GD (52). Tandon et al. found that TSHR 146–165, 160–179, and 202–221 were relevant (53). A logical interpretation of the relation of epitope/DR binding to GD is that strong binding affinity to HLA-DR is related to high efficiency in antigen presentation (7). In fact, an exogenous antigen, such as *Yersinia* that possesses molecular mimicry with TSHR was reported to contribute to development of GD (54). Guarneri and Benvenga reported molecular mimicry between microbial and thyroid autoantigens, and proposed that microbial infection in predisposed subjects might initiate AITDs (55). Furthermore, they reported an *in silico* analysis for amino acid sequence homologies in HLA-DR-binding motifs between some microbial proteins and thyroid autoantigens (TSHR, Tg, and TPO). *Yersinia, Borrelia, Clostridium botulinum, Rickettsia*, and *Helicobacter pylori* were demonstrated to have molecular similarity to these thyroid autoantigens; thus, suggested to be associated with triggering AITD (56). They also reported a patient having HLA-DRB1*03:01 who developed GD possibly by rickettsial infection based on homology with hTSHR/HLA-DR*03:01 binding motif (57). Vita et al. found homology of tumor-associated antigens (NY-ESO-1) used as vaccines, with TSHR, Tg, and TPO in panels of HLA-class I- and class II-binding motifs (58). They concluded that AITD might be elicited by NY-ESO-1 vaccination.

Alternatively, peptides with high-binding affinities to HLA-DR molecules could lead to thymic deletion of the cognate T cells, while those peptides exhibiting moderate binding affinities could escape “negative selection” in the thymus and enter in the circulation and participate in autoimmune disease. Competition between low- and high-risk alleles for binding to TSHR peptides could also affect the development of GD. Due to a higher affinity for specific fragments, protective alleles might prevent binding and presentation of crucial epitopes by high-risk alleles. In addition, certain HLA alleles may not present important epitopes that induce TSHR antibodies. To date, prediction of binding of epitope to HLA-class II is possible as described above, and prediction of binding affinity between epitope and TCR is in development (59).

In mice, a splicing variant of mouse TSHR is related to GD. Endo and Kobayashi described “GD” in mice immunized to the TSHR gene lacking exon 5 (residues 132–157) (60). This observation suggested that exon 5 in TSHR may suppress GD progression, or antibody to residues in exon 5 may contribute to regulate immunity to TSHR. It also suggests that the TSHR-ECD peptide 132–150 (GIFNTGLKMFPDLTKVYST) noted above as a strong HLA binder may not be directly involved in pathogenesis of GD (7).

## Role of Cytokines/Chemokines

For maturation of naïve CD4+ T cells (Th0) in the immunological network, activation of both the TCR and co-stimulatory molecules are necessary (61). APCs, as well as MHC-class II molecules, affect this process. This activation occurs by interaction among epitopes, APCs, and Th0 cells. Subsequently, local cytokine regulation determines whether a Th0 cell will become a Th1 or Th2 cell. The presence of IL-12 and IFN-γ will activate signal transducer and transcription activator 4 (stat 4) and stat 1 signaling pathways, respectively, and promote Th1 cellular differentiation (61). For Th2 differentiation, IL-4 induces GATA3 through the stat-6 signaling pathway. Usually, Th1 cells produce IFN-γ, whereas Th2 cells secrete IL-4, 5, and 13. In GD, activated CD4+ T cells secrete cytokines/chemokines that stimulate B-cells to produce TRAb, and in turn hyperthyroidism occurs. Thus, a preferentially increased Th2/Th1 balance has been reported in GD (62). As IgG1 type of TSAb formation is seen initially in GD, an important role for Th1 was also reported (63). Novel cytokines and chemokines, such as CXCL10 were reported to be related to pathogenesis of GD (64). Increased serum levels of IL-21 (65) and decreased serum IL-7 (66) were also reported in GD. Intriguingly, an association of chromosome 5q23–q33 with AITD suggested an important role of clustered cytokines and other immune modulators encoded in this genetic locus (67). Another subset of Th cells, Th17 cells, was reported to play different roles in mice with different genetic backgrounds (68). Horie et al. genetically immunized NOD-H2(h4) or BALB/c mice with TSHR A-subunit, and found that IL-17 was indispensable for development of Graves’ hyperthyroidism in non-GD-susceptible NOD-H2(h4), but not in GD-susceptible BALB/c mice (68).

## B-Cell Epitope and TRAb in GD

In addition to the T-cell epitopes described above, numerous studies have identified B-cell epitopes of TSHR-ECD in GD. The structure of the Fab fragment of IgG determines the binding affinity to TSHR. TRAb consists of (1) TSAb: thyroid-stimulating antibody, (2) TSBAb: thyroid-stimulation blocking antibody, and (3) neutral TRAb (1, 9, 69). (1) Studies suggest that TSAbs interact with the N-terminal region of the TSHR and transduce a signal through binding sites different from the TSH-binding site (70). R38 in TSHR is the major N-terminal contact residue of TSAb, M22 (71). The TSAb epitopes require involvement of the highly conformational N-terminus of the A-subunit (72). TSAb also preferentially recognize the free A-subunit in animal studies (73). Binding of anti TSHR antibodies to the amino-terminal end of the ECD was confirmed in DR3 transgenic mice (46). TSBAb, usually seen in patients with primary myxedema, recognize the C-terminal region of TSHR-ECD. The antibodies that bound to TSHR residue 381–385 blocked TSHR stimulation by TSH (74).

In contrast to the other glycoprotein hormone receptors, TSHR has ligand-independent (constitutive) activity. Chen et al. found that monoclonal antibody, CS-17, significantly reduces this constitutive activity. This antibody, thus, was considered as “inverse agonist,” but binds to N-terminus of TSHR-ECD, residues 260–289 (75). Rees Smith et al. also showed that antibodies of both stimulating and blocking types bind well to the TSHR (residues 22–260) (76). Neutral TRAb have a linear epitope confined to the cleaved region of TSHR (residues 316–366) (77). By contrast, Hamidi et al. investigated properties of non-stimulatory murine monoclonal antibody, 3BD10. The linear epitope locates in TSHR (residues 31–41) (78). Clinically, Soliman et al. reported that simultaneous recognition of peptides TSHR 158–176 and 248–263 is important for the development of GD (79). In contrast to GD, the functional epitopes of TRAb in HT patients were reported to be uniquely different from those observed in GD (80). A female patient with HT had a blocking type TBII and a weak TSAb. Her blocking type TBII was uniquely reactive with the N-terminal, rather than C-terminal of TSHR-ECD. In addition, her TSAb epitope did not appear to be present solely on the N- or C-terminus of the TSHR-ECD (although the functional epitopes of most TSAb are known to involve the N-terminal region of the receptor) (80). Multimers of TSHR, not monomers, may be required for the maturation of TRAb (81). While little interest is directed to MHC-class II in the development of B-cell epitope in GD, T-cell activation through MHC-class II is indispensable for maturation of TRAb-producing B-cell.

Measurement of TRAb plays an important role in clinical practice. A meta-analysis showed that the overall sensitivity and specificity of the second- and third-generation TRAb assays in GD are 97.1 and 97.4%, and 98.3 and 99.2%, respectively. The likelihood of TRAb-positive individuals to have GD is 1367- to 3420-fold greater compared to that of a TRAb-negative person (82).

## Central and Peripheral Tolerance

In central tolerance, immature T cells with high affinity for autoantigen-derived peptides are deleted in the thymus (7). Regulatory T cells (Tregs) play an important role in suppressing immunogenic T cells in the periphery (peripheral tolerance) (83). Dysfunction of central tolerance in the thymus or Tregs would allow onset of GD. Thyroid autoantigen expression of TSHR, TPO, and Tg in the thymus was not significantly different in different mouse strains (84), suggesting that not only thyroid autoantigen presentation with various MHC molecules but also co-activators or other factors must control self-tolerance. In mouse studies, Tregs are reported not to be involved in TSHR self-tolerance (2). Tregs control the balance between GD and HT (2). Treg numbers in human GD were reported not to be decreased, but Treg function was suggested to be impaired (83, 85). Recent articles further support this Treg functional impairment in several types of CD4+ Treg cells (Foxp3+, CD69+, Tr1) (86).

## Epitope Spreading During Progression of GD

In the course of pathogenic amplification of immunogenic T- and B-cells in GD, “epitope spreading” is frequently seen (50, 69, 87). Intra molecular (TSHR) (69, 88) and inter molecular (Tg, TPO) (2) epitope spreading are observed. The mechanism may relate to developing immunity to host TSHR, and epitope spreading along this antigen (human TSHR to mouse TSHR) (17). Possible reason for T-cell epitope spreading may be the heterogeneity in recognizing thyroid autoantigen (89), or re-arrangements of TCR gene (90). IgG VH gene re-arrangement is known to be associated with B-cell epitope spreading (1, 91). Segundo et al. reported that the occurrence of two distinct types of Thyroid-infiltrating B-lymphocytes. Type 1 B-lymphocytes showed features of marginal zone B-cells, and type 2 B-lymphocytes exhibited a phenotype of germinal center B-cells. They suggested that type 2 might be associated with high titers of TPOAb and not anti-TSHR antibody (92). The role of thyroid-infiltrating B cell in TSHR-related B-cell epitope spreading is yet clear.

## Perspectives on TSHR-Specific Treatments

A novel small molecular TSHR antagonist has been demonstrated to be effective in animal studies as a TSHR-specific treatment for GD (93). TSHR epitope-specific treatments using mutated TSHR peptides were reported to suppress immunogenic reaction of TSHR-ECD in HLA-DR3 transgenic mice immunized to TSHR-ECD protein (46). Peptides in HLA-DR-binding positions 2, 3, 5, and 8 are assumed to be outward facing to stimulate the TCR (8). Therefore, a mutant TSHR peptide was constructed in which the contact of peptide to TCR would be attenuated (46). TSHR peptide 78–94: ISRIYVSID**V**TL**Q**QLES was mutated to TSHR peptide 37m: ISRIYVSID**A**TL**S**QLES, in which DR3-binding motif position 5 was mutated V > A, and position 8 Q > S. 37m was predicted to bind to HLA-DR3, but not bind strongly to TCRs. Both antibody titers to TSHR peptide 78–94, and reaction of splenocytes to TSHR peptide 78–94, were significantly reduced in mice immunized to TSHR peptide 78–94 plus 37m, compared to mice immunized to TSHR peptide 78–94 alone.

The goal of inducing self-tolerance to prevent AITD will require accurate prediction of at-risk individuals together with an antigen-specific therapeutic approach. A transgenic mouse strain having spontaneous TRAb production was developed, and offers further opportunities for investigation of GD *in vivo* (94, 95). As a B-cell-targeted therapy, anti CD20 antibody was reported to be effective for thyroid associated orbitopathy (96). In addition, as well as the acquired immunity described above, innate immunity was suggested to be involved with development of GD (97, 98). Pathogen-associated molecular patterns (PAMPs), danger-associated molecular patterns (DAMPs), and iodide effects on gene expression were reported to be related to innate immune responses (97). The expression of toll-like receptor 4 in thyroid cells may be associated with development of AITDs (98). Thus, specific treatment targeted to innate immunity might be hopeful.

## Conclusion

In the recent years, remarkable progression of research in the mechanism underlying GD was seen. In addition to the function and conformation of TSHR, its binding interaction to HLA-class II molecules and presentation to T cells have been investigated. The relation of TSHR and HLA in terms of TSHR epitope presentation is crucial in development of GD. Numerous studies to identify T- and B-cell epitopes have also demonstrated, including (1) *in silico*, (2) *in vitro*, (3) *in vivo*, and (4) clinical experiments. Dysfunction of central and peripheral tolerance could contribute to development of GD. Although key ideas have been proposed, further investigations are warranted to elucidate precise immunological systems in GD and to establish TSHR epitope-specific treatment.

## Author Contributions

All authors were involved in the preparation and writing of the manuscript.

## Conflict of Interest Statement

The authors declare that the research was conducted in the absence of any commercial or financial relationships that could be construed as a potential conflict of interest.
